# Effects of Increasing CO_2_ Concentration on Crop Growth and Soil Ammonia-Oxidizing Microorganisms in a Fababean (*Vicia faba* L.) and Wheat (*Triticum aestivum* Yunmai) Intercropping System

**DOI:** 10.3390/plants14040516

**Published:** 2025-02-08

**Authors:** Xingshui Dong, Hui Lin, Feng Wang, Songmei Shi, Junwei Ma, Xinhua He

**Affiliations:** 1 State Key Laboratory for Quality and Safety of Agro-Products, Zhejiang Provincial Key Laboratory of Agricultural Microbiomics, Institute of Environment, Resource, Soil and Fertilizer, Zhejiang Academy of Agricultural Sciences, Hangzhou 310021, China; dongxs@zaas.ac.cn (X.D.); wangfeng@zaas.ac.cn (F.W.); majw@zaas.ac.cn (J.M.); 2 National Base of International S&T Collaboration on Water Environmental Monitoring and Simulation in the Three Gorges Reservoir Region and Centre of Excellence for Soil Biology, College of Resources and Environment, Southwest University, Chongqing 400715, China; 2022020@ynau.edu.cn; 3 Department of Land, Air and Water Resources, University of California at Davis, Davis, CA 90616, USA; 4 School of Biological Sciences, University of Western Australia, Perth 6009, Australia

**Keywords:** fababean, wheat, intercropping, elevated CO_2_, ammonia-oxidizing microorganisms

## Abstract

Elevated carbon dioxide (eCO_2_) levels can enhance crop yields but may simultaneously reduce quality, impacting both macronutrient and micronutrient concentrations, and potentially decreasing protein content in cereal grains. This study examined the effects of elevated CO_2_ (eCO_2_) and nitrogen (N) fertilization on crop growth, yield, and soil nitrogen cycling through a glass greenhouse experiment using Eutric Regosol soil. The experimental design incorporated two CO_2_ gradients: ambient CO_2_ (aCO_2_) at approximately 410 ppm during the day and 460 ppm at night, and eCO_2_ at approximately 550 ppm during the day and 610 ppm at night. Additionally, two nitrogen fertilization treatments were applied: no fertilizer (N0) and 100 mg N kg^−1^ dry weight (DW) soil (N100). Crops were cultivated under two cropping systems: the monoculturing of fababean (*Vicia faba* L.) or wheat (*Triticum aestivum* Yunmai) and the intercropping of both species. The results demonstrated that eCO_2_ significantly enhanced the growth and yield of both fababean and wheat, particularly when nitrogen fertilization was applied. Nitrogen fertilizer application did not always enhance crop yield, considering the complexity of nitrogen management under elevated CO_2_ conditions. Furthermore, the intercropping of fababean and wheat presented multiple advantages, including improved crop yields, enhanced soil health, and increased ecosystem services. These findings suggest that intercropping can serve as a sustainable strategy to boost productivity and ecosystem resilience in the face of climate change. The changes in nitrogen application and CO_2_ concentration affect the gene copy number of ammonia-oxidizing bacteria and archaea, which may affect the nitrogen cycling process in soil. There are complex interactions between crop biomass, nitrogen accumulation, transpiration rate, photosynthetic rate and stomatal conductance with soil properties (e.g., pH, organic matter, nitrogen content) and microbial community structure. The interaction between CO_2_ concentration, nitrogen application level and crop intercropping pattern had significant effects on crop growth, soil properties and microbial communities. Future research should prioritize investigating the long-term effects of intercropping on soil productivity and the development of management strategies that optimize the benefits of this cropping system.

## 1. Introduction

Ambient CO_2_ (aCO_2_) is the primary resource for plants to grow and produce biomass through photosynthesis. In the face of intensifying climate change, the concentration of aCO_2_ has been steadily increasing at a rate of 2.0 to 2.4 ppm per year from 2000 to 2019, reaching a peak of 409.9 ppm in 2019—a 5.0% increase [1]. This trend highlights the accelerating climate-related concerns, with projections estimating that CO_2_ concentrations will soar to 700 ppm by the end of the twenty-first century [1]. The rise in CO_2_ concentrations (eCO_2_) has profound environmental impacts, including the intensification of extreme weather phenomena, changes in the distribution of pests and diseases, and effects on crop growth and yield stability. Consequently, these factors increase the uncertainty in agricultural production and threaten food security [2,3,4,5,6].

Fababeans (*Vicia faba* L.), a legume crop with a cultivation history of over 2000 years in China, have made significant contributions to agricultural production and food security through their high productivity and high-quality protein [7]. Research has found that elevated CO_2_ concentrations (eCO_2_) enhance the nitrogen-fixing capacity of leguminous plants, endowing them with greater productivity compared to non-nitrogen-fixing plants [8,9]. Elevated CO_2_ (eCO_2_) is beneficial for C3 crops like fababeans because it enhances photosynthesis, water use efficiency, and yield, particularly under conditions of nitrogen fertilization [10]. Under ample nitrogen fertilization, plants in an elevated CO_2_ (eCO_2_) environment can significantly increase nitrogen uptake and even reduce nitrogen loss from leaves [11]. However, higher nitrogen application rates do not necessarily increase the yield of fababeans [12,13]. These studies indicate that elevated CO_2_ (eCO_2_) has positive effects on the growth and yield of leguminous crops like fababeans, but they also highlight that the relationship between nitrogen fertilization and yield is not linear, necessitating more refined management strategies to optimize crop production. Soil nitrogen-cycling microorganisms play an essential role in promoting nutrient cycling, enhancing plant nutrient uptake, and maintaining soil health through the decomposition of organic matter. In the face of environmental changes such as increased CO_2_ concentrations, these microorganisms may adapt by altering their metabolic pathways and community structures, thereby affecting plant growth and soil fertility. It has been observed that eCO_2_ can lead to a significant reduction in NO_3_^−^-N in the soil, possibly due to increased plant uptake or loss to groundwater and the atmosphere [14,15,16]. The research also found that elevated CO_2_ (eCO_2_) significantly increased the relative abundance of genes associated with nitrogen fixation and denitrification in fababean soil, which may be attributed to the increased carbon input from fallen leaves and rhizosphere secretions [17]. Similarly, in grassland ecosystems, it has been observed that elevated CO_2_ (eCO_2_) stimulates the relative abundance of nitrogen-fixation-related genes. The increase in gene expression has been demonstrated to enhance the growth and nitrogen fixation rate of leguminous plants [18,19].

In the southwestern region of China, the intercropping of fababeans with wheat has shown the ability to enhance crop yields. Studies indicate that while wheat tends to have higher yields under monoculture conditions, the yield of fababeans often sees improvement under intercropping conditions, especially under specific environmental settings [20,21]. Intercropping fababeans with wheat can effectively control the occurrence of plant diseases. Some studies have found that this intercropping pattern can reduce the spread of diseases, thereby improving the overall health and yield of the crops [22]. For instance, intercropping can alleviate the autotoxicity of fababeans and reduce the incidence of certain pathogens [23]. As a nitrogen-fixing plant, fababeans are capable of converting ambient nitrogen into a form usable by plants through rhizobia in their root nodules. This characteristic allows the intercropping of fababeans with wheat to enhance soil nitrogen levels, thereby improving the growth and yield of wheat [24,25]. Long-term intercropping of fababeans and wheat has also been found to improve the physical and chemical properties of the soil. This improvement not only promotes the reconstruction of microbial community structures but also enhances the soil’s water retention and nutrient supply capabilities [25]. Overseas, numerous studies have confirmed the multiple benefits of intercropping fababeans with wheat. For example, in certain regions of Europe, researchers have found that this intercropping system not only increases crop yields but also enhances soil biodiversity, thereby improving ecosystem service functions [22]. Some studies have indicated that intercropping can promote photosynthesis in plants, thereby enhancing the growth rate of fababeans. This effect is not only beneficial for the growth of fababeans but also has a positive impact on the growth of wheat, further promoting the synergistic growth of both crops [26]. By analyzing different planting patterns, researchers have found that intercropping fababeans with wheat is economically viable and can bring higher returns to farmers. Especially in regions where resources are relatively scarce, the intercropping model can make more efficient use of land and resources [22,27]. The research also emphasizes the importance of management strategies. By employing well-designed intercropping practices, it is possible to effectively balance the resource competition and complementary relationships between crops. For instance, adjusting planting density and row spacing, along with employing appropriate fertilization and irrigation strategies, can further enhance the benefits of intercropping [21,27]. Although current research has shown many benefits of fababeans and wheat intercropping, there are still some research gaps and future directions to explore, e.g., elevated CO_2_ effects of intercropping on soil health and productivity; since different varieties of fababeans and wheat may behave differently in intercropping, choosing the right variety is crucial; emerging agricultural technology and management tools can help farmers better implement intercropping and improve their economic and ecological benefits. More policy support and scientific outreach are needed to promote the spread and application of intercropping techniques to help farmers increase yields and incomes. We designed an experiment to determine the potential and advantage of intercropping fababean and wheat under elevated CO_2_ levels. This experiment detected the change of growth productivity and photosynthesis character for the fababean and wheat and the soil ammonia-oxidizing microbial. We hypothesize that (1) eCO_2_ or nitrogen supply enhanced the growth and nitrogen concentration of fababean and wheat; (2) eCO_2_ or nitrogen supply changed the soil ammonia-oxidizing microbial. This research will further deepen the understanding of this intercropping model to provide theoretical support and practical guidance for sustainable agricultural development. By better understanding these interactions, we can more effectively predict and respond to the potential impacts of future climate change on agroecosystems.

## 2. Results

### 2.1. Effects of Nitrogen and eCO_2_ on Growth Indexes of Intercropping Fababean and Wheat

**Figure 1:**
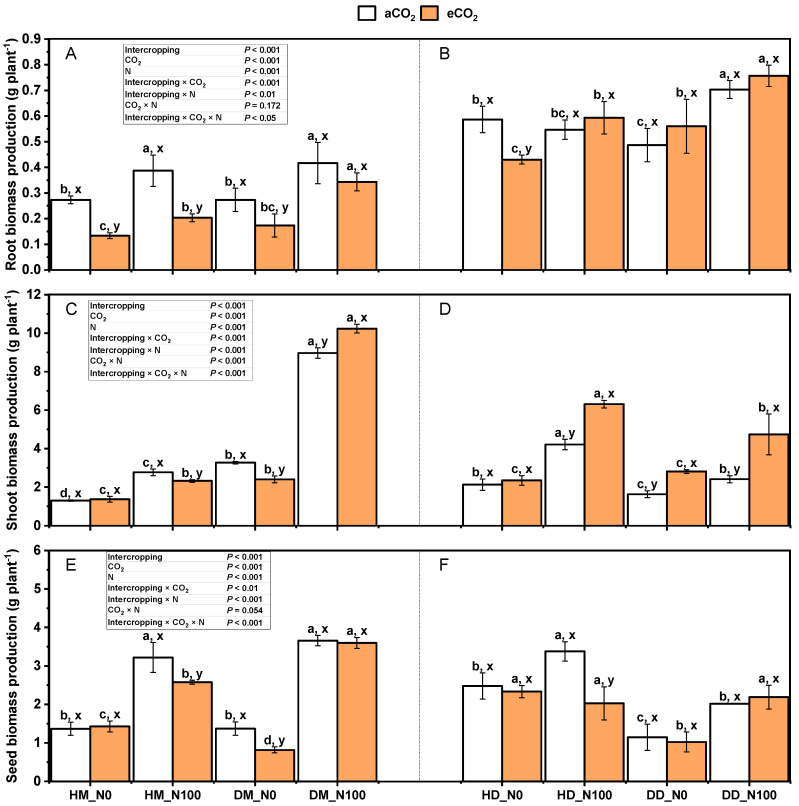
Effects of eCO_2_ and N supply on root biomass (**A**,**B**); stem (stem and leaf) biomass (**C**,**D**); seed biomass (**E**,**F**) of 5-month-old fababean and wheat at harvest stage. Data are means ± SE (*n* = 3). Lowercase letters above the bars indicate a significant difference between N supplies (a, b) for the same CO_2_ treatment and between CO_2_ concentrations (x, y) for the same N treatment *p* < 0.05. Abbreviations: aCO_2_, ambient CO_2_; eCO_2_, elevated CO_2_; N0, no N supply; N100, 100 mg N kg^−1^ DW soil.

Under aCO_2_ and eCO_2_ treatments, N100 significantly increased seed yield, above-ground biomass, and root biomass of monoculture (DM) and intercropping wheat (HM) (Figure 1A,C,E) (*p* < 0.05). However, the root and seed yields of monocropping (DD) and intercropping fababean (HD) did not show a similar uniform increase trend in wheat, except for a significant increase trend in stem leaves (*p* < 0.05) (Figure 1B,D,F). Under the treatment of aCO_2_ and eCO_2_, N100 significantly increased the root and seed biomass of monocultural fababean (HD). Under aCO_2_ and eCO_2_ treatments, the stem and leaf biomass of monocultures increased more than that of intercropping wheat stimulated by N100, which was three times the increase in stem and leaf biomass of intercropping wheat (Figure 1C). This phenomenon was reversed for fababeans, and the increase in stem and leaf biomass in monocultural fababeans was less affected by N100 than that in intercropping fababeans (Figure 1D). Intercropping × CO_2_, intercropping × N, intercropping × CO_2_ × N, and intercropping × CO_2_ × N had significant effects on stem and leaf biomass. Intercropping × CO_2_, intercropping × N, and intercropping × CO_2_ × N interactions had significant effects on root and seed biomass.

**Figure 2:**
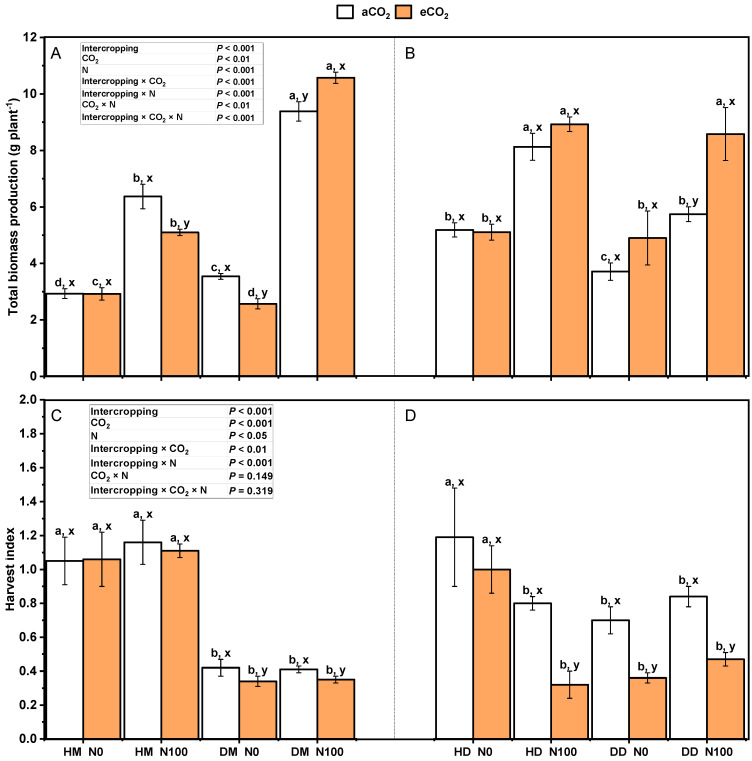
Effects of eCO_2_ and N supply on (**A**,**B**) the total biomass; (**C**,**D**) Harvest index = seed yield/stem biomass of fababean and wheat at 5 months of age. Data are means ± SE (*n* = 3). Lowercase letters above the bars indicate a significant difference between N supplies (a, b) for the same CO_2_ treatment and between CO_2_ concentrations (x, y) for the same N treatment *p* < 0.05. Abbreviations: aCO_2_, ambient CO_2_; eCO_2_, elevated CO_2_; N0, no N supply; N100, 100 mg N kg^−1^ DW soil.

Under the treatment of aCO_2_ and eCO_2_, N100 significantly increased the total biomass of wheat in the fababean–wheat system, in which the total biomass of monoculture wheat was the most stimulated by N100, and the growth rate was three times that of intercropping wheat. Under the treatment of aCO_2_ and eCO_2_, N100 significantly increased the total biomass of fababeans in the fababean–wheat system. Under N0 and N100 treatments, the total biomass of monocropping fababeans was lower than that of intercropping fababeans, which was significantly reduced under aCO_2_ conditions (Figure 2A,B). The CO_2_ and N inputs do change the harvest index (Figure 2C,D). Under N0 and N100 treatments, eCO_2_ significantly reduced the yield index of monoculture wheat but had no significant effect on the yield index of intercropping wheat. For fababeans treated with N0 and N100, CO_2_ significantly reduced the harvest index of fababeans in the fababean–wheat system, except for intercropped fababeans treated with N0. Under aCO_2_ and eCO_2_ treatment, the harvest index of intercropping fababean was more affected by N100 than that of monoculture fababean. N100 significantly decreased the harvest index of intercropping fababean but had no significant effect on the harvest index of monoculture fababean. Under all CO_2_ and N treatments, the yield index of monoculture wheat was significantly lower than that of intercropping wheat. Under the N0 treatment, the harvest index of monocultural fababeans was significantly lower than that of intercropping fababeans (Figure 2C,D). Intercropping × CO_2_, intercropping × N, intercropping × CO_2_ × N, and intercropping × CO_2_ × N had significant effects on the total biomass. The interaction of intercropping × CO_2_ and intercropping × N had significant effects on the harvest index.

**Figure 3:**
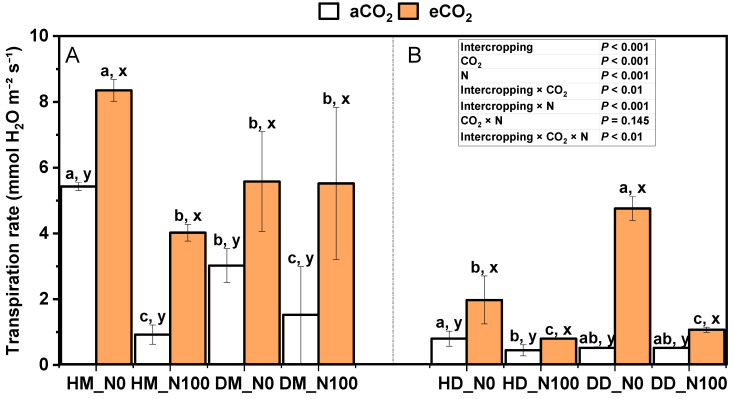
Effects of eCO_2_ and N supply on transpiration rate (**A**,**B**) during the booting stage of 5-month-old fababean and wheat. Data are means ± SE (*n* = 3). Lowercase letters above the bars indicate a significant difference between N supplies (a, b) for the same CO_2_ treatment and between CO_2_ concentrations (x, y) for the same N treatment *p* < 0.05. Abbreviations: aCO_2_, ambient CO_2_; eCO_2_, elevated CO_2_; N0, no N supply; N100, 100 mg N kg^−1^ DW soil.

Under aCO_2_ and eCO_2_ treatments, N100 significantly reduced the transpiration rate of intercropping wheat (Figure 3A). Under aCO_2_ treatment, N100 significantly reduced the transpiration rate of monoculture wheat. Under the treatment of aCO_2_ and eCO_2_, N100 significantly reduced the transpiration rate of fababean in the fababean–wheat system (Figure 3B). N100 significantly decreased the transpiration rate of intercropping fababean under aCO_2_ treatment but had no significant effect on the transpiration rate of monoculturing fababean. Under the treatment of N0 and N100, eCO_2_ significantly increased the transpiration rate of wheat and fababean in the fababean–wheat system but significantly decreased the transpiration rate of intercropping fababean. Under the same nitrogen and CO_2_ treatment, the transpiration rate of intercropping wheat and intercropping fababean was higher than that of monoculturing wheat and monoculturing fababean. Intercropping × CO_2_, intercropping × N, and intercropping × CO_2_ × N had significant effects on transpiration rate.

### 2.2. Effects of Nitrogen and eCO_2_ on Photosynthetic Parameters of Intercropping Fababean and Wheat Leaves

**Figure 4:**
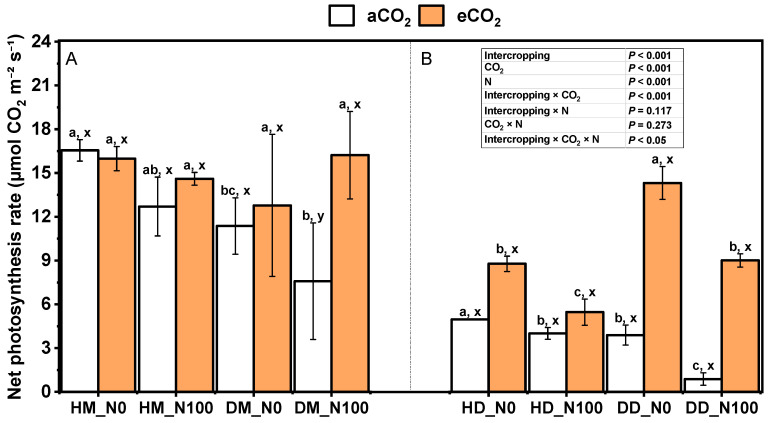
Effects of eCO_2_ and N supply on net photosynthetic rate (**A**,**B**) during the booting stage of 5-month-old fababean and wheat. Data are means ± SE (*n* = 3). Lowercase letters above the bars indicate a significant difference between N supplies (a, b) for the same CO_2_ treatment and between CO_2_ concentrations (x, y) for the same N treatment *p* < 0.05. Abbreviations: aCO_2_, ambient CO_2_; eCO_2_, elevated CO_2_; N0, no N supply; N100, 100 mg N kg^−1^ DW soil.

Under aCO_2_ and eCO_2_ treatments, N100 reduced the net photosynthetic rate of intercropping wheat, but not significantly (Figure 4A). Under the treatment of aCO_2_ and eCO_2_, N100 had no significant effect on the photosynthetic rate of monoculture wheat. Under the treatment of aCO_2_ and eCO_2_, N100 significantly reduced the net photosynthetic rate of fababeans in the Douma system (Figure 4B). Under aCO_2_ treatment, N100 had a higher effect on the net photosynthetic rate reduction of fababean under monocropping mode. Under N0 and N100 treatments, eCO_2_ increased the net photosynthetic rate of wheat under monocropping and intercropping modes (except intercropping N0 treatment), and significantly increased the net photosynthetic rate of small fababeans under the fababean–wheat system, and the increased value of the net photosynthetic rate of monocultural fababeans was greater than that of intercropping fababeans. Under the same nitrogen and CO_2_ treatment, the net photosynthetic rate of intercropping wheat was higher than that of monocropping wheat (except N100), while for fababean, the net photosynthetic rate of monocropping fababean was significantly higher than that of intercropping fababean. The interaction of intercropping × CO_2_ and intercropping × CO_2_ × N had significant effects on net photosynthetic rate.

**Figure 5:**
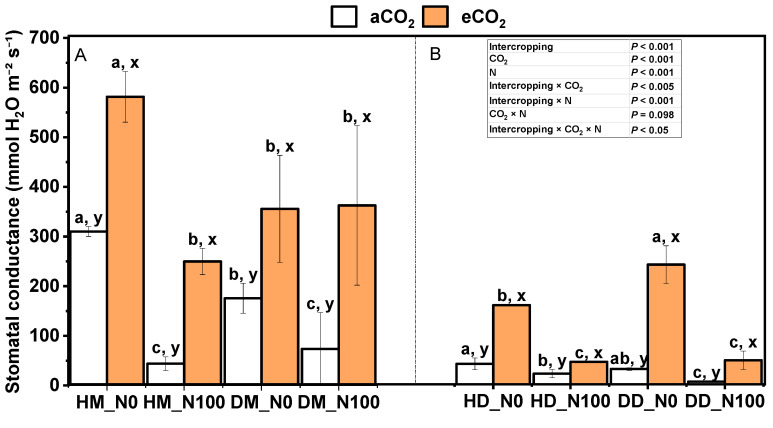
Effects of eCO_2_ and N supply on stomatal conductance (**A**,**B**) during the booting stage of 5-month-old fababean and wheat. Data are means ± SE (*n* = 3). Lowercase letters above the bars indicate a significant difference between N supplies (a, b) for the same CO_2_ treatment and between CO_2_ concentrations (x, y) for the same N treatment *p* < 0.05. Abbreviations: aCO_2_, ambient CO_2_; eCO_2_, elevated CO_2_; N0, no N supply; N100, 100 mg N kg^−1^ DW soil.

Under aCO_2_ and eCO_2_ treatments, N100 significantly reduced the stomatal conductance of intercropping wheat (Figure 5A). Under aCO_2_ and eCO_2_ treatments, N100 had no significant effect on the stomatal conductance of monocropping wheat, but N0 significantly decreased the impact. Under aCO_2_ and eCO_2_ treatments, N100 significantly reduced the stomatal conductance of fababeans in the Douma system (Figure 5B). Under N0 and N100 treatments, eCO_2_ increased the stomatal conductance of wheat and fababean under monocropping and intercropping modes. Under the same nitrogen and CO_2_ treatment, the stomatal conductance of intercropping wheat was significantly higher than that of monocropping wheat. The interaction of intercropping × CO_2_, intercropping × N, and intercropping × CO_2_ × N had significant effects on stomatal conductance.

**Figure 6:**
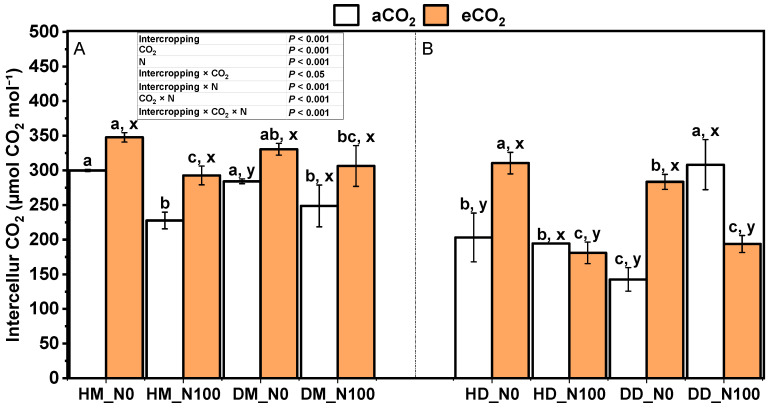
Effects of eCO_2_ and N supply on intercellular CO_2_ concentration (**A**,**B**) during the booting stage of 5-month-old fababean and wheat. Data are means ± SE (*n* = 3). Lowercase letters above the bars indicate a significant difference between N supplies (a, b) for the same CO_2_ treatment and between CO_2_ concentrations (x, y) for the same N treatment *p* < 0.05. Abbreviations: aCO_2_, ambient CO_2_; eCO_2_, elevated CO_2_; N0, no N supply; N100, 100 mg N kg^−1^ DW soil.

Under aCO_2_ and eCO_2_ treatments, N100 significantly reduced the intercellular CO_2_ concentration in wheat in both intercropping and monoculture modes (Figure 6A). Under aCO_2_ and eCO_2_ treatments, N100 significantly reduced the intercellular CO_2_ concentration of fababeans in the fababean–wheat system, except for the results under aCO_2_ treatment of fababeans alone, where the intercellular CO_2_ concentration was significantly increased under the influence of N100 (Figure 6B). Under N0 and N100 treatment, eCO_2_ significantly increased the intercellular CO_2_ concentration in wheat under monocropping and intercropping modes. Under N0 treatment, eCO_2_ significantly increased the intercellular CO_2_ concentration of small fababeans in the fababean–wheat system. Under N100 treatment, eCO_2_ significantly reduced the intercellular CO_2_ concentration in fababean under monocropping and intercropping modes. Under the same nitrogen and CO_2_ treatment, there was no significant change in intercellular CO_2_ concentration between intercropping wheat and monoculture wheat. For fababeans, under aCO_2_ and eCO_2_ treatments, the intercellular CO_2_ concentration of monocultural fababeans under N0 treatment was significantly higher than that of intercropping fababeans, while there was no uniform change trend in the pattern of bean and wheat system under N100 treatment. Intercropping × CO_2_, intercropping × N, intercropping × CO_2_ × N, and intercropping × CO_2_ × N interactions had significant effects on the intercellular CO_2_ concentration.

**Figure 7:**
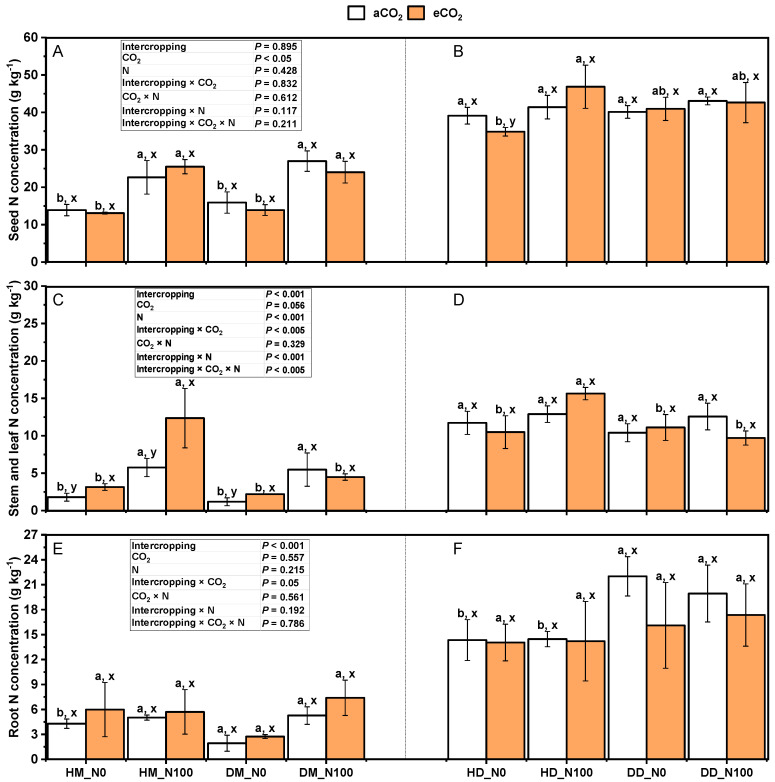
Effects of eCO_2_ and N supply on tissue N concentrations and accumulations in seed (**A**,**B**), stem and leaf (**C**,**D**), and root (**E**,**F**) of 5-month-old fababean and wheat at harvest. Data are means ± SE (*n* = 3). Lowercase letters above the bars indicate a significant difference between N supplies (a, b) for the same CO_2_ treatment and between CO_2_ concentrations (x, y) for the same N treatment *p* < 0.05. Abbreviations: aCO_2_, ambient CO_2_; eCO_2_, elevated CO_2_; N0, no N supply; N100, 100 mg N kg^−1^ DW soil.

Under the treatment of aCO_2_ and eCO_2_, N100 significantly increased the nitrogen concentrations of wheat seeds, stems, leaves, and roots (Figure 7A,C,E) (*p* < 0.05). However, nitrogen concentrations in stem seeds and stem leaves of intercropping fababean did not increase significantly (*p* < 0.05), and nitrogen concentrations in seeds, stem leaves, and roots of monocropping fababean did not show a similar uniform increase trend in wheat (Figure 7B,D,F). Under N0 and N100 treatments, eCO_2_ significantly increased nitrogen concentrations in wheat seeds, stem leaves, and roots under intercropping mode but had no significant effects on nitrogen concentrations in seed, stem leaves, and roots of fababean in monocultures of wheat and the fababean–wheat system. Under the same nitrogen application and CO_2_ treatment, there was no significant change in nitrogen concentration between the seeds, stem leaves, and roots of intercropping wheat fababean and monocropping wheat. Except for wheat above-ground parts under N100 treatment and wheat roots under N0 treatment, the intercropping mode was significantly higher than that under the monocropping mode. Intercropping × CO_2_, intercropping × N, intercropping × CO_2_ × N, and intercropping × CO_2_ × N had no significant effects on the nitrogen concentration of wheat fababean seeds in the fababean–wheat system. Intercropping × CO_2_, intercropping × N, and intercropping × CO_2_ × N had significant effects on the nitrogen concentration in the stem and leaves of wheat fababean in the fababean–wheat system. Intercropping × CO_2_, intercropping × N, intercropping × CO_2_ × N, and intercropping × CO_2_ × N had no significant effects on root nitrogen concentration of wheat fababean in the fababean–wheat system.

### 2.3. Effects of Nitrogen and eCO_2_ on Total Nitrogen in Various Parts of Intercropping Fababean and Wheat

**Figure 8:**
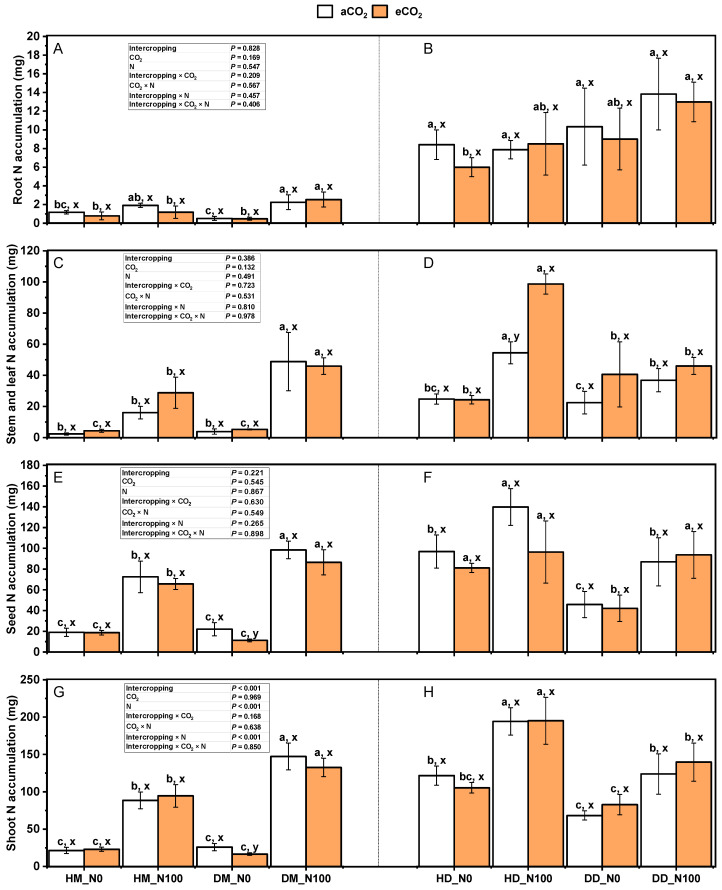
Effects of eCO_2_ and N supply on tissue N accumulations in roots (**A**,**B**), stems (stems and leaves) (**C**,**D**), seeds (**E**,**F**), and above-ground tissues (**G**,**H**) of 5-month-old fababean and wheat at harvest. Data are means ± SE (*n* = 3). Lowercase letters above the bars indicate a significant difference between N supplies (a, b) for the same CO_2_ treatment and between CO_2_ concentrations (x, y) for the same N treatment *p* < 0.05. Abbreviations: aCO_2_, ambient CO_2_; eCO_2_, elevated CO_2_; N0, no N supply; N100, 100 mg N kg^−1^ DW soil.

Under the treatment of aCO_2_ and eCO_2_, N100 significantly increased the nitrogen accumulation in wheat seeds, stem leaves, above-ground parts, and roots (Figure 8A,C,E) (*p* < 0.05). However, for fababean, nitrogen accumulation in seeds, stem leaves, and above-ground parts of intercropping fababean showed a significant increasing trend (*p* < 0.05), while nitrogen accumulation in seeds, stem leaves, above-ground parts, and roots of monocropping fababean did not show a similar uniform increasing trend in wheat (Figure 8B,D,F,H). Under the treatment of N0 and N100, eCO_2_ increased the nitrogen accumulation in stem leaves and the above-ground part of wheat under intercropping mode (*p* > 0.05). Nitrogen accumulation in wheat seeds and roots was decreased (*p* > 0.05). Under N0 and N100 treatments, eCO_2_ increased the nitrogen accumulation in the stem and leaves of fababean (*p_HD_N100_* < 0.05) but decreased the nitrogen accumulation in the seed of fababean under intercropping mode (*p* > 0.05). Under the same nitrogen application and CO_2_ treatment, nitrogen accumulation in intercropping wheat seeds, stem leaves, above-ground parts, and roots was lower than that in monocultural wheat seeds, stem leaves, above-ground parts, and roots (*p_N100_* < 0.05). Nitrogen accumulation in seeds, stems, and leaves of intercropping fababean was lower than that in seeds, stems, and leaves of monoculturing fababean (*p* < 0.05), stems and leaves of stem (*p_N100_* < 0.05), and ground (*p_N100_* < 0.05). Intercropping × CO_2_, intercropping × N, intercropping × CO_2_ × N, and intercropping × CO_2_ × N had no significant effects on nitrogen accumulation in seeds, stem leaves, above-ground parts, and roots of the wheat–fababean system.

### 2.4. Effects of Nitrogen and eCO_2_ Application on pH, Organic Matter, Total Nitrogen, NH_4_^+^-N and NO_3_^−^-N in Intercropped Soils

**Figure 9:**
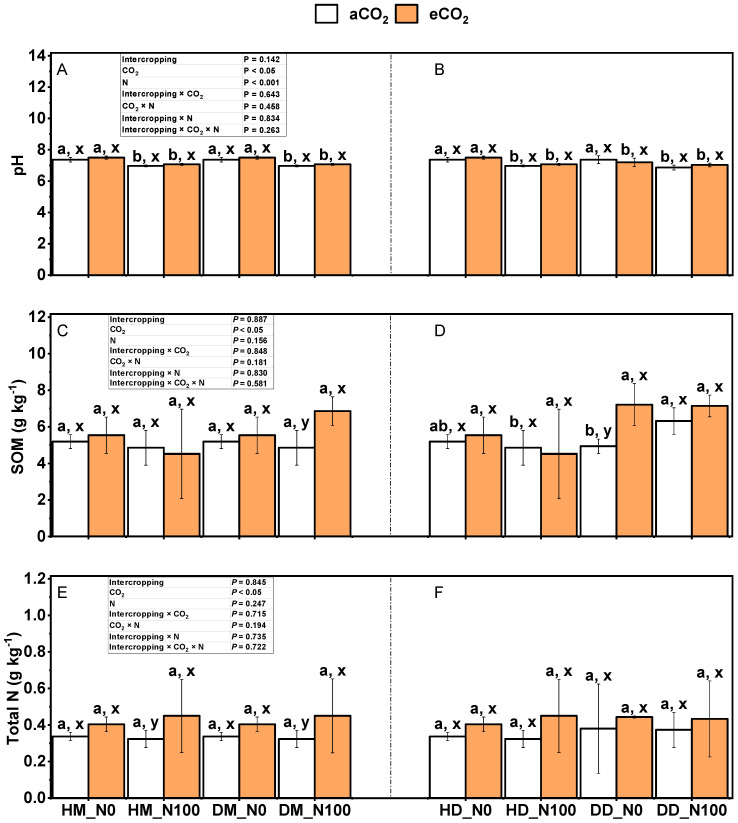
Effects of eCO_2_ and N supply on soil pH (**A**,**B**); soil organic matter (**C**,**D**); soil total nitrogen (**E**,**F**) at harvest of 5-month-old fababean and wheat. Abbreviations: aCO_2_, ambient CO_2_; eCO_2_, elevated CO_2_; N0, no N supply; N100, 100 mg N kg^−1^ DW soil. Data are means ± SE (*n* = 3). Lowercase letters above the bars indicate a significant difference between N supplies (a, b) for the same CO_2_ treatment and between CO_2_ concentrations (x, y) for the same N treatment *p* < 0.05. Abbreviations: aCO_2_, ambient CO_2_; eCO_2_, elevated CO_2_; N0, no N supply; N100, 100 mg N kg^−1^ DW soil.

**Figure 10:**
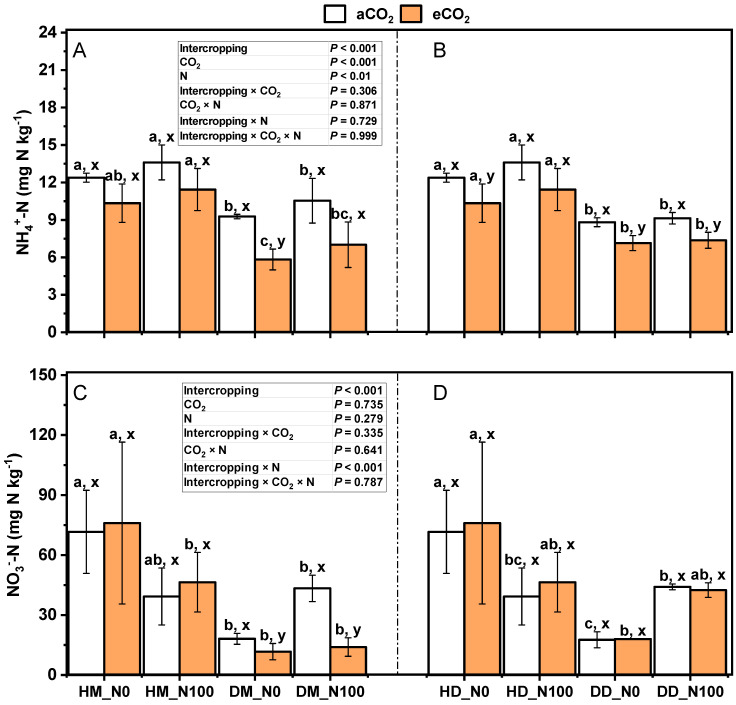
Effects of eCO_2_ and N supply on NH_4_^+^-N (**A**,**B**); NO_3_^−^-N (**C**,**D**) at harvest of 5-month-old fababean and wheat. Abbreviations: aCO_2_, ambient CO_2_; eCO_2_, elevated CO_2_; N0, no N supply; N100, 100 mg N kg^−1^ DW soil. Data are means ± SE (*n* = 3). Lowercase letters above the bars indicate a significant difference between N supplies (a, b) for the same CO_2_ treatment and between CO_2_ concentrations (x, y) for the same N treatment *p* < 0.05. Abbreviations: aCO_2_, ambient CO_2_; eCO_2_, elevated CO_2_; N0, no N supply; N100, 100 mg N kg^−1^ DW soil.

The effects of CO_2_ and nitrogen application on soil pH, soil organic matter, and soil total nitrogen during the harvest of wheat fababean in the fababean–wheat system are shown in Figure 5, Figure 6, Figure 7, Figure 8, Figure 9 and Figure 10. Under aCO_2_ and eCO_2_ treatments, N100 significantly reduced the pH in wheat and fababean soil (Figure 8A,B) (*p* < 0.05), but had no significant effect on soil organic matter and soil total nitrogen (Figure 8C–F) (*p* > 0.05). Under N0 and N100 treatments, eCO_2_ had no significant effects on soil pH, organic matter, and total nitrogen of wheat and fababean soil (*p* > 0.05), except that eCO_2_ significantly increased the organic matter in monocultural fababean soil under N0 treatment (*p* < 0.05). Under the same nitrogen application and CO_2_ treatment, planting mode had no significant effects on soil pH, organic matter, and total nitrogen of wheat and fababean (*p* > 0.05), except that under monocropping mode, soil organic matter of fababean was significantly higher than that under intercropping. Intercropping × CO_2_, intercropping × N, intercropping × CO_2_ × N, and intercropping × CO_2_ × N had no significant effects on soil pH, organic matter, and total nitrogen in wheat and fababean soil.

The effects of CO_2_ and nitrogen application on soil NH_4_^+^-N and NO_3_^−^-N during the harvest of wheat fababeans in the bean–wheat system are shown in Figure 10. Under aCO_2_ and eCO_2_ treatments, N100 decreased NO_3_^−^-N in the soil of intercropping wheat–fababean (*p* > 0.05) and increased NO_3_^−^-N in the soil of monoculture wheat–fababean (*p* > 0.05) (no significant change in NO_3_^−^-N in the soil of monoculture wheat under eCO_2_ treatment). Under N0 and N100 treatments, eCO_2_ decreased NH_4_^+^-N in wheat and fababean soil (*p* > 0.05), increased NO_3_^−^-N in wheat and fababean soil (*p* > 0.05), and decreased NO_3_^−^-N in wheat and fababean soil under monocultures (*p_DM_* < 0.05). Under the same nitrogen application and CO_2_ treatment, NH_4_^+^-N and NO_3_^−^-N in intercropping wheat and fababean soil were significantly higher than those in monoculture fababean soil (*p* < 0.05). The interaction of intercropping × CO_2_, CO_2_ × N, and intercropping × CO_2_ × N had no significant effects on NH_4_^+^-N and NO_3_^−^-N of wheat fababean soil in the bean–wheat system while intercropping × N had significant effects on NO_3_^−^-N of wheat fababean soil in the bean–wheat system.

### 2.5. Effects of Nitrogen and eCO_2_ on the Gene Copy Number of Ammonia-Oxidizing Bacteria and Ammonia-Oxidizing Archaea in Soil

**Figure 11:**
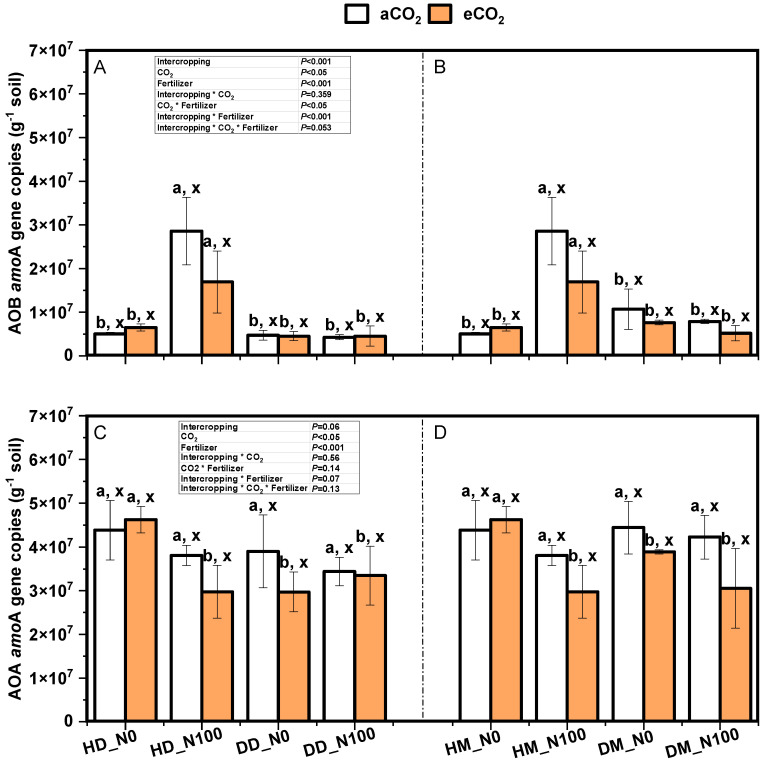
Gene copies of soil ammonia-oxidizing bacteria (AOB) in fababean (**A**) and wheat (**B**), and gene copies of soil ammonia-oxidizing archaea (AOA) in fababean (**C**) and wheat (**D**) under different nitrogen and CO_2_ treatments. Data are means ± SE (*n* = 3). Lowercase letters above the bars indicate a significant difference between N supplies (a, b) for the same CO_2_ treatment and between CO_2_ concentrations (x, y) for the same N treatment *p* < 0.05. Abbreviations: aCO_2_, ambient CO_2_; eCO_2_, elevated CO_2_; N0, no N supply; N100, 100 mg N kg^−1^ DW soil.

Under aCO_2_ and eCO_2_ treatments, the gene copy number of ammonia-oxidizing bacteria and ammonia-oxidizing archaea were significantly affected by nitrogen application in intercropping mode but not in the soil of fababean and wheat under monoculture mode. Under N0 and N100 treatments, eCO_2_ treatment reduced the gene copy number of ammonia-oxidizing bacteria and ammonia-oxidizing archaea, except for intercropping without nitrogen fertilizer, but had no significant effect (Figure 11). Except for intercropping × CO_2_ and intercropping × CO_2_ × N, intercropping × N and CO_2_ × N interactions had significant effects on the gene copy number of ammonia-oxidizing bacteria. Intercropping × CO_2_, intercropping × N, intercropping × CO_2_ × N, and intercropping × CO_2_ × N had no significant effect on copy number of genes of ammonia-oxidizing archaea.

### 2.6. Pairwise Spearman Correlation Matrix of AOA and AOB Abundance, Environmental Factors, Plant Growth Indexes, and Leaf Photosynthetic Parameters Under Nitrogen Application and eCO_2_ Treatment

**Figure 12:**
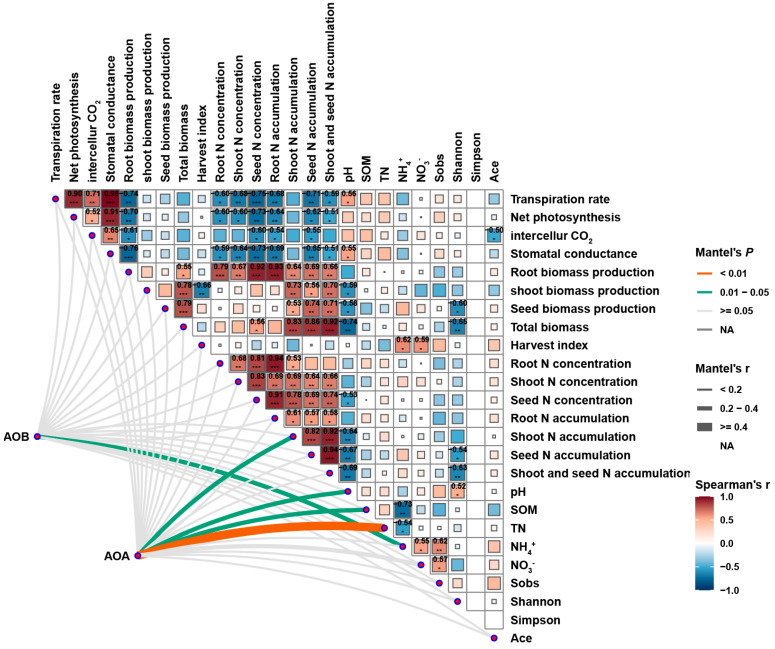
Pairwise Spearman’s correlation matrix of AOA, AOB, environmental factors, plant growth characters, and photosynthesis characters. A color gradient indicates Spearman’s correlation coefficients. *: indicates the significance level (*p*-value). *: *p* < 0.05 (significant). **: *p* < 0.01 (very significant). ***: *p* < 0.001 (extremely significant). No marker: No significant association (*p* ≥ 0.05).

The Spearman correlation matrix in Figure 12 showed that AOA was significantly positively correlated with above-ground nitrogen accumulation, soil pH, soil organic matter, and soil total nitrogen, while AOB was significantly positively correlated with soil ammonium nitrogen (Mantel’s *p* < 0.05). The biomass and total biomass of root, above-ground and seed were positively correlated with nitrogen accumulation of root, above-ground and seed. Nitrogen accumulation and total nitrogen concentration in roots, above-ground parts, and seeds were negatively correlated with leaf transpiration rate, net photosynthetic rate, intercellular carbon dioxide, and stomatal conductance. There was a significant positive correlation between harvest index and soil ammonium nitrogen and nitrate nitrogen. There was a significant positive correlation between pH and soil nitrogen cycling-related microbial Alpha diversity index (Figure 12).

## 3. Discussion

### 3.1. Response of Fababean and Wheat Biomass to Nitrogen and eCO_2_ Application in Intercropping System

CO_2_ shapes plant growth and development by influencing photosynthesis, respiration, and other physiological processes in plants (Figure 1). In general, increasing ambient CO_2_ concentration is thought to have a positive effect on plant biomass [28]. Studies have shown that an increase in CO_2_ concentration typically leads to a 30% to 50% increase in net photosynthesis in plants [29]. Plants can convert light energy into chemical energy through photosynthesis, and CO_2_ is an indispensable raw material in this process. As CO_2_ concentration increases, the photosynthetic rate of plants generally increases, thus promoting the growth of biomass [30]. When the CO_2_ concentration increased from 350 μmol/mol to 560 μmol/mol, the dry weight of the plant (including the main leaves, main stems, branches, and roots) increased significantly, indicating that a high-CO_2_ environment can improve plant growth [31]. This growth-promoting effect is prevalent in different plant species, including cereal and bean crops. Although C4 crops (such as corn and sugarcane) behave similarly to C3 crops (such as wheat and peas) under high-CO_2_ conditions, the photosynthetic efficiency of C4 crops may respond differently in high-CO_2_ environments [29]. This means that biomass generally increases at elevated CO_2_ concentrations for both C3 and C4 crops, but the exact magnitude of the increase may vary by crop type [32]. With global climate change, rising temperatures may also work with increasing CO_2_ concentrations to affect crop biomass. Higher temperatures can lengthen the growing season, which may further increase crop biomass [33,34,35,36]. However, excessive temperatures can also lead to stress responses that affect the overall health and yield of crops [21]. Higher CO_2_ concentrations often boost photosynthesis in plants but do not always lead to an increase in biomass, especially in C3 plants such as broad beans and wheat, which can be affected by a combination of factors [37]. Although eCO_2_ can increase the photosynthetic rate of C3 plants in the short term, in the long term, plants may show a photosynthetic adaptation phenomenon, that is, the photosynthetic efficiency gradually decreases. eCO_2_ increases plant demand for nutrients such as nitrogen and phosphorus. If the supply of these nutrients in the soil is insufficient, the biomass accumulation of plants may be limited. eCO_2_ inhibits plant respiration, but this inhibition may vary depending on plant species and environmental conditions [38]. For some C3 plants, inhibition of respiration may result in limited plant growth. eCO_2_ generally reduces stomatal conductance and reduces transpiration, thereby increasing water use efficiency [39]. However, if soil water is insufficient, this change may not fully compensate for the lack of water, which in turn affects plant biomass. eCO_2_ may lead to the accumulation of metabolites in the plant body, such as increased starch and tannin content and decreased soluble sugar and protein content [40]. These changes may affect the normal physiological functions of plants, thereby limiting the accumulation of biomass.

Increasing the supply of nitrogen fertilizer can further contribute to the growth of plant biomass in a high-CO_2_ environment (Figure 2B). This is because nitrogen is essential for protein synthesis and growth in plants. It has been found that nitrogen supply, acting together with CO_2_ concentration, can significantly increase crop biomass [41,42,43,44]. Soil nitrogen is an important nutrient element in ecosystems and plays a key role in plant growth, soil health, and ecological balance. Nitrogen is present in soil in various forms, mainly including organic nitrogen compounds, ammonium ions (NH_4_^+^), and nitrate ions (NO_3_^−^). The transformation of these different forms of nitrogen into the soil is crucial for plant growth and ecosystem function. Organic nitrogen is the main form of nitrogen in soil and is derived from plant residues, animal excreta, and microbial metabolites. These organic nitrogen compounds are converted into nitrogen in the soil by mineralization, which can be absorbed by plants. Their presence not only provides the nitrogen needed by plants but also promotes the activities of microorganisms related to the soil nitrogen cycle, which helps to improve soil fertility [16]. Ions are another major form of nitrogen and are usually released through fertilization or mineralization of soil organic matter. NH_4_^+^ is a direct source of nitrogen absorbed by plants that can be quickly utilized by plant roots. The presence of ammonium ions is very important for maintaining plant growth and physiological processes, as it is involved in a variety of biochemical reactions [29,45]. Nitrate is another important form of nitrogen in soil and is usually converted from ammonium ions by nitration. NO_3_^−^ is one of the most commonly used nitrogen sources in plants, and its conversion rate is fast and it can be quickly absorbed by plants. The presence of nitrate has an important effect on the growth and development of plants, especially in the case of sufficient nitrogen application, which can significantly increase the biomass of plants [46]. Nitrogen in the soil exists in many forms and is constantly being transformed. Nitrogen fixation is essential for increasing the nitrogen content of the soil and promoting plant growth [47].

An increase in CO_2_ concentration has a significant positive effect on crop biomass, especially under appropriate nitrogen application. However, with the intensification of climate change, the increase in CO_2_ alone is not enough to ensure the healthy growth of crops, and the impact of nitrogen, temperature, and other environmental factors must be considered comprehensively. Therefore, in future agricultural management, more integrated measures are needed to ensure sustainable agricultural production in a high-concentration CO_2_ environment.

### 3.2. Response of Photosynthetic Parameters of Fababean and Wheat Leaves to Nitrogen and eCO_2_ Application in Intercropping System

The photosynthetic response of fababean and wheat leaves under different carbon dioxide concentrations is an important research field in plant physiology. Photosynthesis is the process by which plants convert carbon dioxide and water into organic matter and oxygen through light energy. Carbon dioxide is one of the raw materials of photosynthesis, and its concentration change will directly affect the growth and yield of plants. Studies have shown that increasing carbon dioxide concentrations generally significantly increases the photosynthetic rate of plants [48]. C3 plants, such as fababeans and wheat, are significantly more efficient at photosynthesis under high-CO_2_ conditions (Figure 4). For example, under the right water supply conditions, high carbon dioxide concentrations can significantly enhance the photosynthetic capacity of leaves, thus promoting plant growth and increasing yield [49]. High CO_2_ concentrations are also associated with an increase in water use efficiency (WUE), which, in the presence of sufficient water, increases the plant’s photosynthetic rate while closing the plant’s stomata, thereby reducing water loss through evaporation [50]. This phenomenon is particularly important for plants grown in drought conditions, which require more efficient use of limited water resources [51]. The rate of adjustment of photosynthetic efficiency plays an important role in the carbon gain and yield of crops. Different plants respond to changes in carbon dioxide concentration at different rates, and plants that adapt quickly can make better use of light energy and carbon dioxide in a changing environment, thereby increasing their growth rate and yield. For example, it was found that the flag leaf of wheat can rapidly adjust its photosynthetic efficiency under the action of high carbon dioxide and nitrogen fertilizer [52]. Different plant species also respond differently to carbon dioxide concentrations. The study showed that even in the same high-carbon dioxide environment, the photosynthetic carbon dioxide absorption capacity of fababeans and wheat is significantly different, and this difference may be related to the physiological characteristics of the plant, the photosynthetic mechanism, and the need for light and water [53]. Under high-CO_2_ conditions, the photosynthetic physiological parameters of plants (such as chlorophyll content, stomatal conductance, and photophosphorylation rate) usually change significantly (Figure 3 and Figure 5). Studies have shown that the chlorophyll content and photosynthetic efficiency of fababean have been improved in a high carbon dioxide environment, thus promoting the synthesis of photosynthetic products. The increase in carbon dioxide concentration combined with the application of nitrogen fertilizer can further enhance the photosynthetic capacity of plants [32]. The stomatal conductance of broad beans and wheat generally decreases under elevated carbon dioxide concentrations, but under certain conditions, stomatal conductance may increase or exhibit different response mechanisms. At higher temperatures, stomatal conductance may increase. Studies have shown that the stomatal conductance of C3 plants, such as wheat and broad beans, is sensitive to the interaction of temperature and carbon dioxide concentration [54]. When temperatures rise, stomatal conductance may increase to regulate leaf temperature, even at high CO_2_ concentrations. Under low light intensity conditions, stomatal conductance may increase. This is because low light intensity reduces the rate of photosynthesis, and stomata need to be more open to maintain an adequate supply of carbon dioxide [55]. Under sufficient soil moisture conditions, stomatal conductance may increase. This is because when water is sufficient, plants do not need to reduce water loss by closing their stomata, allowing them to remain more open [56]. After prolonged exposure to high carbon dioxide concentrations, plants may adapt to environmental changes by adjusting stomatal density or stomatal behavior. Some studies have found that plants may exhibit a short-term increase in stomatal conductance at high CO_2_ concentrations, followed by a gradual decrease [57]. The response of different plants to the increase in carbon dioxide concentration is different. For example, certain plant varieties or genotypes may exhibit higher stomatal conductance to maintain higher photosynthetic rates [58]. Nitrogen is an important nutrient element for plant growth, which can promote the growth of leaves and the synthesis of photosynthetic pigments, thereby improving photosynthetic efficiency. Under the condition of a full supply of nitrogen fertilizer, high concentrations of carbon dioxide can promote the photosynthetic rate of fababeans and wheat, thereby increasing their growth and yield [59]. By regulating the concentration of carbon dioxide, we can improve the photosynthetic capacity of plants, enhance the water use efficiency, and further enhance the growth potential of plants through proper coordination with nitrogen fertilizer.

### 3.3. Response of Nitrogen Concentration in Various Parts of Fababean and Wheat to Nitrogen Application and eCO_2_ in Intercropping System

In high-CO_2_ environments, the transpiration of plants may be reduced, which affects nutrient distribution (Figure 7 and Figure 8). In this case, while biomass may increase, the concentration of certain nutrients (such as nitrogen, magnesium, and phosphorus) may decrease, which may negatively affect the nutritional value of the crop [60]. The interaction mechanism of carbon dioxide (CO_2_) concentration and nitrogen (N) supply level is particularly important when studying the biomass response of fababean and wheat in intercropping systems. As the concentration of carbon dioxide in the atmosphere increased, the photosynthetic efficiency of fababean as a C3 crop increased significantly. This increase not only enhanced the photosynthetic rate of fababean but also improved its water use efficiency and yield [61]. Under the condition of adequate nitrogen application, the growth of fababean showed stronger adaptability and better utilization of environmental resources, thus promoting the increase in its biomass [62]. The increased CO_2_ concentration promotes photosynthesis, allowing fababeans to produce more organic matter when light and water conditions are suitable. At high concentrations of CO_2_, fababeans show better water use efficiency, which is crucial for their growth, especially under drought conditions [61]. Nitrogen is a key nutrient element for plant growth and development, especially in intercropping systems, where nitrogen supply levels directly affect the biomass of fababeans and wheat. In the case of insufficient nitrogen application, fababeans can reduce the demand for external nitrogen fertilizer through the biological nitrogen fixation mechanism, which not only reduces production costs but also reduces greenhouse gas emissions. The roots of fababeans can improve the physical properties of the soil and increase the nutrient retention capacity of the soil [63]. The biological nitrogen fixation ability of fababean enables it to maintain good growth even under the condition of insufficient nitrogen application and then support the root system of wheat to penetrate the soil, improve the soil structure and fertility, and make wheat grow better in the same soil [32]. In the intercropping system, the interaction mechanism between fababean and wheat is complicated. The nitrogen fixation ability of fababeans increases the nitrogen content of the soil, which helps the growth of wheat [13]. At the same time, the growth of wheat can also provide a certain shade for fababeans and reduce the evaporation of water in the early growth of fababeans [64]. The intercropping of fababeans and wheat showed a complementary effect, with fababeans providing nitrogen sources while wheat provided growth conditions for fababeans through its growth [52]. Although the two are complementary in resource utilization, there is also a competitive relationship, especially in access to water and sunlight [49]. Therefore, at different CO_2_ concentrations and nitrogen levels, the growth performance of the two may be different [65]. Under different CO_2_ concentration and nitrogen application levels, the biomass responses of fababean and wheat showed significant changes. High concentrations of CO_2_ and high nitrogen application usually result in optimal growth of both, while low CO_2_ and low nitrogen application may result in growth restriction [51]. Fababean had the highest photosynthetic efficiency and water use efficiency, and the biomass increased significantly. At the same time, wheat also benefited from the nitrogen source of fababean, showing a better growth state (Figure 1 and Figure 4). In this environment, the growth of fababeans is severely limited, and the biological nitrogen fixation capacity is insufficient, resulting in wheat also unable to obtain enough nitrogen, which affects its growth and yield [66]. To better understand the interaction mechanism between fababeans and wheat in the intercropping system, future studies can reveal the biological response mechanism of fababeans and wheat under different environmental conditions through genomics and metabolomics studies. A long-term field experiment was conducted to observe the long-term effects of different CO_2_ concentrations and nitrogen application levels on the growth of fababean and wheat. Finally, an ecological model was established to simulate the growth dynamics of fababean and wheat under different environmental conditions to provide theoretical support for agricultural management.

### 3.4. Response of Soil Ammonia-Oxidizing Microorganisms to Nitrogen and eCO_2_ Application in Intercropping System

Ammonia-oxidizing microorganisms are an important microbial community in soil and are mainly responsible for the oxidation of ammonia to nitrite and nitrate, a process that is essential for the soil nitrogen cycle and plant nutrition [36]. With global warming and increased agricultural activities, carbon dioxide (CO_2_) emissions have risen significantly, which has had a profound impact on the activities of microorganisms associated with the soil nitrogen cycle. The study showed that high CO_2_ concentrations had significant effects on the community structure and function of AOA and AOB [67] (Figure 11). According to relevant studies, when CO_2_ concentrations rise, the abundance and nitriding capacity of ammonia-oxidizing microorganisms in surface soil increase significantly [68]. This change is mainly due to the increase in CO_2_, which changes the chemical properties of soil and the growth environment of microorganisms. When CO_2_ concentration increases, the abundance of ammonia-oxidizing archaea increases more significantly than that of ammonia-oxidizing bacteria, which may be related to the ability to adapt to high-concentration CO_2_ environments [69]. This difference suggests that ammonia-oxidizing archaea may play a more important role in future climate change [67]. The high concentration of CO_2_ promoted the ammonia oxidation reaction, which led to the increase in the formation of nitrite and nitrate in the soil, thus affecting the whole nitrogen cycle. This process not only affects the nitrogen supply of plants but also may lead to nitrogen loss, affecting soil fertility and ecological balance [70].

The increase in CO_2_ concentration is usually accompanied by an increase in soil temperature and a change in moisture [71]. These changes in environmental conditions will directly affect the metabolic activities of microorganisms. For example, an increase in temperature can accelerate the ammonia oxidation process, thereby increasing the activity of ammonia-oxidizing microorganisms [72]. The increase in CO_2_ may lead to the acceleration of the decomposition rate of organic matter in the soil, thus releasing more nutrients and promoting the growth and reproduction of ammonia-oxidizing microorganisms. At the same time, the application of nitrogen fertilizer will further enhance the ammonia concentration in the soil and provide more substrates for ammonia-oxidizing microorganisms [44]. In an environment of elevated CO_2_ concentration, the competitive relationship between ammonia-oxidizing microorganisms and other microorganisms related to the soil nitrogen cycle may change. Some microorganisms may take advantage of a high-CO_2_ environment due to their strong adaptability, and change the composition of soil nitrogen cycling-related microbial communities [9]. Ammonia-oxidizing microorganisms play a key role in soil ecosystems, and their activities not only affect the nitrogen cycle but are also closely related to other ecological processes: the ammonia-oxidizing process is one of the main pathways for the production of nitrous oxide (N_2_O). N_2_O is a potent greenhouse gas with a global warming potential 298 times greater than CO_2_. Therefore, changes in the activity of ammonia-oxidizing microorganisms directly affect the emission of N_2_O in the soil, which in turn affects global climate change [73]. Ammonia oxidizing microorganisms affect plant growth and crop yield by regulating soil nitrogen status. In environments with high concentrations of CO_2_ and nitrogen fertilizer application, the activities of these microorganisms may cause nitrogen loss in the soil and reduce soil quality [74]. The effects of long-term CO_2_ increase on the ammoxidation microbial communities and their ecological functions are not fully understood, and long-term field trials and laboratory studies are needed. Crop rotation systems may have different effects on the response of ammoxidation microorganisms, especially in systems where multiple crops are symbiotic, where microbial interactions may be more complex. Determining how to optimize the activity of ammonia-oxidizing microorganisms through reasonable fertilization and soil management measures to reduce greenhouse gas emissions and improve soil health is an important direction of future research. As global CO_2_ concentrations continue to rise, understanding the response mechanisms of these microorganisms and their ecological implications has important scientific value for developing sustainable agricultural management strategies and addressing climate change.

In the fababean and wheat intercropping system, the reaction mechanism of ammonia-oxidizing microorganisms to nitrogen fertilizer application mainly includes the basic process of the nitrogen cycle, the effect of nitrogen fertilizer application on the structure and function of ammonia-oxidizing microorganisms, and the interaction with soil environmental factors. Nitrogen is an important nutrient essential for plant growth. In soil, nitrogen conversion is mainly achieved through ammonia oxidation and nitrification processes. Nitrogen fertilizer application can change the community structure of ammonia-oxidizing microorganisms in soil. The results show that the application of nitrogen fertilizer can promote the proliferation of ammonia-oxidizing bacteria, especially in the case of high nitrogen fertilizer application. This is because nitrogen fertilizer provides an adequate source of ammonia and stimulates the activity of ammonia-oxidizing bacteria. For example, in diazot-applied soils, the proportion of AOB in the bacterial community increased significantly, indicating a more pronounced response to nitrogen fertilizer [9]. After the application of nitrogen fertilizer, the concentration of ammonia in the soil increased, which directly promoted the rate of ammonia oxidation. The metabolic activity of ammonia-oxidizing microorganisms such as AOB and AOA is enhanced in a high nitrogen environment, which leads to the acceleration of the ammonia oxidation rate. This phenomenon has been observed in different soil types, especially when nitrogen sources such as ammonium salts and urea are applied [75]. Soil physical and chemical properties (such as pH, soil moisture, organic matter content, etc.) also affect the response of ammonia-oxidizing microorganisms to nitrogen fertilizer. For example, in acidic soils, the activity of ammonia-oxidizing archaea is relatively high, and the application of nitrogen fertilizer may lead to an increase in their relative abundance, while in neutral or alkaline soils, ammonia-oxidizing bacteria may be more active [76]. In addition, changes in soil oxygen concentration after nitrogen fertilizer application will also affect the ammonia oxidation capacity of microorganisms, especially under the condition of high soil moisture, which may lead to the emergence of anaerobic conditions, thus inhibiting the activity of some ammonia-oxidizing microorganisms. Long-term application of nitrogen fertilizer may lead to the adaptation of ammonia-oxidizing microorganisms. With the continuous application of nitrogen sources, microorganisms may gradually adapt to the high nitrogen environment and show stronger ammonia oxidation capacity. However, this adaptation may also be accompanied by a loss of microbial diversity, and long-term fertilization may lead to the degradation or disappearance of certain microbial populations [77].

Studies have shown that the fababean–wheat intercropping system can significantly increase the abundance and activity of ammonia-oxidizing microorganisms [78]. In intercropping, the root exudates of fababean provided rich organic matter for the ammonia-oxidizing microorganisms, which not only promoted the growth and reproduction of the microorganisms but also improved the nitrogen availability of the soil. Compared with monocultural systems, intercropping can make more efficient use of soil nitrogen resources, thereby improving crop yield and quality. In addition, the diversity of intercropping systems also helps maintain the stability of microbial communities, making them more resilient to environmental changes. The functional optimization of ammonia-oxidizing microorganisms is not only reflected in the increase in their abundance but also in the change in their community structure. The study found that the relative abundance of ammonia-oxidizing archaea (AOA) increased in the fababean–wheat intercropping system, suggesting that different types of microorganisms can form more complex networks in their interactions, thereby improving the efficiency of the nitrogen cycle [79]. The increase in carbon dioxide concentration and the application of nitrogen fertilizer had significant effects on the function of ammonia-oxidizing microorganisms (Figure 11 and Figure 12). Studies have shown that elevated CO_2_ concentrations can stimulate plant photosynthesis and increase the amount of root exudates, which in turn provides more organic matter for ammoxidation microorganisms [4]. With the increase in CO_2_ concentration, the growth rate of plants accelerated, and the biomass of roots increased. More root exudates provide a rich source of nutrients for the ammonia-oxidizing microorganisms, thus promoting their growth and activity. This change not only increased the abundance of ammonia-oxidizing microorganisms but also enhanced their ammonia-oxidizing ability, which in turn increased the availability of nitrogen. Nitrogen fertilizer application is an important means to increase nitrogen supply in soil. However, excessive application of nitrogen fertilizer may lead to the inhibition of ammonia-oxidizing microorganisms, resulting in nitrogen loss and environmental pollution. Therefore, a rational nitrogen fertilizer application strategy should be the key to promoting the optimization of ammonia-oxidizing microbial function.

## 4. Methods and Materials

The experiment was carried out in a glass greenhouse with a basin (25 cm in diameter) containing 5 kg of soil in November 2018, which was classified as Eutric Regosol (FAO soil classification system), developed from the purplish-red mudstone and shale of the Shaximiao Formation in the Jurassic period and had the following basic chemical properties: The pH value was 7.4 (1:2.5 soil to water ratio), organic matter content was 9.00 g kg^−1^, total nitrogen content was 0.53 g kg^−1^, NH_4_^+^-N content was 7.81 mg kg^−1^, NO_3_^−^-N content was 16.47 mg kg^−1^, and four plants were planted in each pot. The weather here was subtropical monsoon humid climate. The average annual temperature is 18.3 °C, and the average annual precipitation is 1105.4 mm.

Two CO_2_ gradients were set in the experimental environment: ambient CO_2_ concentration was about 410 (day)/460 (night) ± 30 ppm (aCO_2_), and high concentration CO_2_ concentration was about 550 (day)/610 (night) ± 30 ppm (eCO_2_); there are two gradients of facility fertilizer treatments: no fertilizer (N0) and nitrogen fertilizer (N100, 100 mg N kg^−1^ DW soil); two planting modes were set up: monoculturing monoculture of fababean (*Vicia faba* L.) (DD) or wheat (*Triticum aestivum* Yunmai) (DM) and intercropping of fababean (two plants each pot) (HD) and wheat (two plants each pot) (DM), each treatment had three replications.

### 4.1. Plant and Soil Sampling Methods

At the time of the fababean and wheat harvest (about 5 months of growth), plant and soil samples were collected while wearing disposable gloves. First, we used a tape measure to measure plant height from the ground to the very top of the plant and recorded it. The plant was further divided into roots, stems, leaves, and seeds. The plant samples were dried in an oven at 70 °C for 72 h. We recorded the number of seeds per plant. The dry weight of seed, stem, and root biomass was recorded.

After removing the excess soil around the basin, the rhizosphere soil was finally collected by shaking the root. Ten soil samples were collected from different locations in the same pot to create a mixed sample. We removed any plant material or debris from the soil sample. The collected soil samples were packaged in sterile ziplock bags, transported to the laboratory in a portable refrigerator (−18 °C), and stored at −80 °C for soil DNA extraction. A portion of the soil samples were ground through 2 mm and 0.25 mm screens and air-dried for soil physical and chemical properties analysis.

### 4.2. Measurement Method of Photosynthetic Parameters

Photosynthetic parameters were measured during the flowering stage of fababean. On a sunny morning from 8:30 to 11:30 a.m. in April 2019, we carefully selected four to six fully unfolded compound leaves from the stem tips. During the grain booting stage of wheat, photosynthetic parameters were measured. Wheat plants with similar growth were selected, and the flag leaves of their main stem were used for measuring photosynthetic parameters, which were conducted between 8:30 and 11:30 a.m. on the sunny morning of April 2019. Measurements were made using the Li-6400XT portable Photosynthesis System (Li-Cor Inc., Lincoln, NE, USA) with a built-in red and blue light source. The light intensity was set to 1000 µmol m^−2^ s^−1^. Blade temperature was controlled at 20–25 °C, and VPD was controlled in the range of 1–4 kPa. Under aCO_2_ conditions, the CO_2_ concentration in the reference chamber for N0 and N100 treatments was maintained at 410 µmol mol^−1^, while under eCO_2_ conditions, the CO_2_ concentration for the same treatment was maintained at 560 µmol mol^−1^. Parameters recorded included net photosynthetic rate (Pn), stomatal conductance (Gs), intercellular CO_2_ concentration (Ci), and transpiration rate (E).

### 4.3. Methods for Testing Physical and Chemical Properties of Plants and Soils

The physical and chemical properties of soil were analyzed by standard methods [80]. Soil pH was determined with a soil–water ratio (weight/volume) of 1:2.5 using the LE438 composite electrode meter (Mettler Toledo Instruments Co., Ltd., Shanghai, China). The content of soil organic matter was determined by the potassium dichromate external heating method, and the content of soil total nitrogen was determined by the Kjeldahl nitrogen determination method. Soluble inorganic nitrogen (ammonium nitrogen-NH_4_^+^-N and nitrate nitrogen-NO_3_^−^-N) from soil was extracted by the Bremner method [81]. The total nitrogen content of plants was determined by indigo phenol blue colorimetry, which involves boiling the test solution with a mixture of sulfuric acid and hydrogen peroxide and then measuring it at a wavelength of 690 nm (UV-1800, Telescope (Shanghai) Co., Ltd., Shanghai, China).

### 4.4. Quantitative PCR Method

Real-time fluorescence quantitative PCR was used to determine the abundance of amoA genes in AOB and AOA. amoA gene amplification primers for AOB and AOA and their gene sequences are shown in Table 1. The PCR reaction system was 20 μL, consisting of l0 μL SYBR Premix Ex TaqTM, 0.5 μL upstream primer (10 μM), 0.5 μL downstream primer, 2 μL DNA template, and 7 μL ultra-pure sterile water. The AOB amoA gene PCR amplification procedure was: 94 °C, 5 min; 32 × (94 °C, 45 s; 56 °C, 45 s; 72 °C, 1 min); 72 °C, 10min; hold at 4 °C. AOA amoA gene was amplified by ordinary PCR for 94 °C, 5 min. Next, 32 × (94 °C, 45 s; 53 °C, 45 s; 72 °C, 90 s); 72 °C, 10 min; hold at 4 °C. 

### 4.5. Statistical Analysis

SPSS 19.0 (SPSS Inc., Chicago, IL, USA) was used to analyze the differences between different treatments, and a two-factor analysis of variance was used. Data (mean ± standard deviation, *n* = 3) were tested by Duncan’s multiple range test (*p* < 0.05). Correlation coefficients were analyzed using GraphPad Prism 10.2.0 (GraphPad Software, Boston, MA, USA). We used Origin Pro 2021(OriginLab, Northampton, MA, USA) and R 4.3.3 (Posit Software, Boston, MA, USA) for mapping.

## 5. Conclusions

Under high CO_2_ concentrations (eCO_2_) and ambient CO_2_ concentrations (aCO_2_), nitrogen application (N100) significantly increased the seed yield, above-ground biomass, and root biomass of wheat in the fababean–wheat system. Nitrogen application had positive effects on the root and seed yield of fababean in the fababean–wheat system, but this effect was more significant in wheat. Intercropping wheat showed higher stem biomass than monoculture wheat after N application, but the transpiration rate was decreased due to N application. Conversely, fababeans showed a stronger response to nitrogen application, both in stem biomass and transpiration rate, during monocropping than during intercropping. At high CO_2_ concentrations, nitrogen application decreased the net photosynthetic rate and stomatal conductance of wheat and fababean, suggesting that there may be downward regulation of photosynthesis. Nitrogen application reduced the transpiration rate of wheat, but the effect on fababeans depended on whether the crop was monocropping or intercropping. Nitrogen application decreased the pH value of wheat and fababean soil but had no significant effect on soil organic matter and total nitrogen. Nitrogen application decreased NO_3_^−^-N content in intercropped wheat and fababean soil, while eCO_2_ treatment increased NO_3_^−^-N content in soil.

## Figures and Tables

**Table 1:** Primer sequences of ammonia-oxidizing microorganisms (AOB, AOA).

Gene Name	Primer Sequence (5′-3′)	Fragment Length
AOB *amo*A	Upstream primer *amo*A-1F GGGGTTTCTACTGGTGGT	491 bp
	Downstream primer *amo*A-2R CCCCTCKGSAAAGCCTTCTTC	
AOA *amo*A	Upstream primer Arch-*amo*AF STAATGGTCTGGCTTAGACG	635 bp
	Downstream primer Arch-*amo*AR GCGGCCATCCATCTGTATGT	

## Data Availability

Data are contained within the article.
